# Combined neuromuscular electrical stimulation and transcutaneous spinal direct current stimulation increases motor cortical plasticity in healthy humans

**DOI:** 10.3389/fnins.2022.1034451

**Published:** 2023-01-13

**Authors:** Tadaki Koseki, Daisuke Kudo, Kaito Yoshida, Mitsuhiro Nito, Keita Takano, Masafumi Jin, Shigeo Tanabe, Toshiaki Sato, Hiroshi Katoh, Tomofumi Yamaguchi

**Affiliations:** ^1^Graduate School of Health Sciences, Yamagata Prefectural University of Health Sciences, Yamagata, Japan; ^2^Department of Physical Therapy, Yamagata Prefectural University of Health Sciences, Yamagata, Japan; ^3^Department of Anatomy and Structural Science, Yamagata University School of Medicine, Yamagata, Japan; ^4^Faculty of Rehabilitation, School of Health Sciences, Fujita Health University, Toyoake, Japan; ^5^Department of Occupational Therapy, Yamagata Prefectural University of Health Sciences, Yamagata, Japan; ^6^Department of Physical Therapy, Faculty of Health Science, Juntendo University, Tokyo, Japan

**Keywords:** afferent input, corticospinal projection, neural plasticity, motor cortical excitability, spinal excitability

## Abstract

**Introduction:**

Neuromuscular electrical stimulation (NMES) induces neural plasticity of the central nervous system (CNS) and improves motor function in patients with CNS lesions. However, the extended stimulus duration of NMES reduces its clinical applicability. Transcutaneous spinal direct current stimulation (tsDCS), which increases afferent input, may enhance the effects and reduce the stimulus duration of NMES. This study investigated the excitability of the motor cortex, somatosensory cortex, and spinal motor neurons after the combined stimulation of NMES and tsDCS.

**Methods:**

Among the 55 participants in this study, 24 were allocated to experiment 1, 15 to experiment 2, and 16 to experiment 3. They received intervention for 20 min on different days: (1) NMES combined with tsDCS (NMES + tsDCS), (2) NMES combined with sham tsDCS (NMES + sham tsDCS), and (3) sham NMES combined with tsDCS (sham NMES + tsDCS). NMES was delivered to the right common peroneal nerve at 25 Hz with the intensity at 120% of the motor threshold. For tsDCS, the cathodal electrode was positioned on the thoracic 10th–12th vertebral levels, and the anodal electrode was located on the right shoulder. The stimulus intensity was 2.5 mA. In experiment 1, motor evoked potentials (MEPs) and short-latency intracortical inhibition (SICI) were measured by transcranial magnetic stimulation up to 60 min after stimulation. The spinal motor neurons’ excitability was assessed by recording the posterior root muscle reflex (PRMR) induced *via* transcutaneous spinal cord stimulation in experiment 2, and the primary somatosensory cortex excitability was evaluated by recording the somatosensory evoked potentials (SEPs) in experiment 3 up to 15 min after stimulation.

**Results:**

Compared to before the stimulation, NMES + tsDCS significantly increased MEP for 60 min or more, and significantly decreased SICI immediately after. Conversely contrast, the PRMR significantly decreased immediately after, and SEPs were unchanged.

**Discussion:**

These results suggest that simultaneous afferent inputs from different stimulus positions critically induce primary motor cortex plasticity. The combined stimulation of NMES with tsDCS may facilitate the development of a new neurorehabilitation technique.

## 1. Introduction

Neural plasticity plays a crucial role in improving motor function and motor performance after stroke and spinal cord injury (Nudo et al., 1996; Jurkiewicz et al., 2007; Yamaguchi et al., 2016; Christiansen and Perez, 2018; Fujiwara, 2020). Therefore, rehabilitation strategies that enhance plastic changes in the cerebral cortex and spinal circuits’ excitability are strongly desired for patients with central nervous system (CNS) lesions.

One strategy used to induce neural plasticity is neuromuscular electrical stimulation (NMES), which increases sensory input and enhances the excitability of the sensorimotor cortex and spinal circuits in humans (Khaslavskaia et al., 2002; Knash et al., 2003; Perez et al., 2003; Shin et al., 2008; Fujiwara et al., 2009; Takahashi et al., 2018). However, NMES requires over 30 min of electrical stimulation to induce neural plasticity (Chipchase et al., 2011). This prolonged time interferes with its clinical application. A Cochrane review (Pollock et al., 2014) reported that 30–60 min of active rehabilitation per day provides significant benefits toward the functional recovery of patients with stroke. Thus, prolonged stimulus time prevents active rehabilitation.

However, NMES has been recommended as an adjunctive therapeutic modality to improve motor function and voluntary movement in patients with stroke (Knutson et al., 2015). Previous studies have suggested that NMES combined with voluntary contraction (Khaslavskaia and Sinkjaer, 2005; Yamaguchi et al., 2012; Takahashi et al., 2018) and non-invasive stimulation techniques, such as transcranial direct current stimulation (Rizzo et al., 2014; Yamaguchi et al., 2016) or repetitive transcranial magnetic stimulation (Yamaguchi et al., 2018; Du et al., 2022), may boost neural plasticity and recovery of motor function.

Recently, transcutaneous spinal direct current stimulation (tsDCS) was proposed to induce neural plasticity in the motor cortex and spinal circuits in humans (Bocci et al., 2014, 2015a; Yamaguchi et al., 2020). tsDCS leads to neural membrane depolarization or hyperpolarization of spinal dorsal axons, inducing neural plasticity of motor and sensory systems in a polarity-specific manner (Cogiamanian et al., 2008; Winkler et al., 2010). Moreover, tsDCS promotes the voluntary movement of leg muscles (Berry et al., 2017; Yamaguchi et al., 2020). In particular, cathodal tsDCS consistently facilitates motor cortex, motor-related pathway, and voluntary-motor output (Bocci et al., 2014, 2015a,b; Murray et al., 2018; Powell et al., 2018a; Yamaguchi et al., 2020; Therkidsen et al., 2022). In addition, the neural plasticity of the motor cortex is influenced by the dose-dependent response of the sensory input through electrical stimulation (Powell et al., 2018b; Insausti-Delgado et al., 2021). Therefore, we hypothesize that the combination of NMES with cathodal tsDCS may induce an enduring increase in the sensorimotor cortex and spinal circuits’ excitability by the summation of sensory input to the sensorimotor cortex *via* NMES and the membrane potential of spinal dorsal axons depolarization *via* tsDCS. In the present study, to investigate the combined effects of NMES and tsDCS on the neural plasticity of CNS, we measured the excitability of the motor cortex, somatosensory cortex, and spinal circuits in healthy adults. The results of this investigation may provide a foundation for a new therapeutic strategy in patients with CNS lesions.

## 2. Materials and methods

### 2.1. Participants

A total of 55 healthy volunteers, with 24 (aged 24 ± 4 years; 24 males) allocated to experiment 1, 15 (aged 25 ± 4 years; 15 males) to experiment 2, and 16 (24 ± 4 years; 16 males) to experiment 3, were enrolled in this study. The sample size was determined based on the effect size (g = 0.50) of a previous study investigating the effect of tsDCS on corticospinal excitability (Murray et al., 2018). The sample size was determined based on a previous study investigating the effect of NMES on somatosensory cortex excitability (Schabrun et al., 2012). The participants had no history of neurological and/or musculoskeletal disorders. All participants provided written informed consent before participation. The study was approved by the Ethical Review Board of the Yamagata Prefectural University of Health Science in Japan (approval number: 2103-17) and was performed according to the ethical standards of the Declaration of Helsinki.

### 2.2. General experimental design

This study comprised three experiments designed to evaluate the combined effects of NMES with tsDCS on the neural plasticity of motor and sensory systems in humans. We employed a single-blinded (participants), randomized crossover experimental design for each of the three experiments. In experiment 1, the combined effects of NMES and tsDCS on the excitability of the motor cortex were evaluated by recording the motor evoked potential (MEP) and short-interval intracortical inhibition (SICI) induced *via* transcranial magnetic stimulation (TMS). In experiment 2, we investigated the combined effect of NMES and tsDCS on spinal excitability was evaluated by recording the posterior root muscle reflex (PRMR) induced *via* transcutaneous spinal cord stimulation (tSCS). Lastly, in experiment 3, the combined effect of NMES and tsDCS on the excitability of the primary somatosensory cortex (S1) was evaluated by recording the somatosensory evoked potential (SEP). All participants received NMES and tsDCS during three separate 20-min sessions on different days: (1) combined stimulation of NMES and cathodal tsDCS (NMES + tsDCS), (2) NMES combined with sham tsDCS (NMES + sham tsDCS), and (3) sham NMES combined with cathodal tsDCS (sham NMES + tsDCS). The session order was randomly assigned by a computer-generated list. To avoid carryover effects from the previous intervention, washout intervals of three days or more were inserted between sessions.

#### 2.2.1. NMES

Neuromuscular electrical stimulation was delivered to the right common peroneal nerve (CPN) *via* bipolar stimulus electrodes (1.5 cm diameter, 3.0 cm inter-electrode distance) connected to Neuropack MEB-2200 (Nihon Kohden, Tokyo, Japan). NMES consisted of a train of 20 pulses at 25 Hz with a 50% duty cycle for 20 min (Knash et al., 2003; Andrews et al., 2013). The stimulation intensity was 120% of the motor threshold (MT) in the right tibialis anterior (TA) muscle with a pulse width of 0.2 ms (McKay et al., 2002; Knash et al., 2003; Andrews et al., 2013). The MT was defined by the minimum stimulation intensity that evoked a 100 μV M-wave in the TA. The sham NMES was performed with the same electrode position and stimulation device, but without an active electrode (Guo and Kang, 2018).

#### 2.2.2. tsDCS

Transcutaneous spinal direct current stimulation was delivered by using a NeuroConn DC Stimulator Plus (Neurocare group GMBH, Germany) through two carbon rubber electrodes (5 cm × 7 cm) with electroconductive gel (Signagel, Parker Laboratories, USA) to keep impedance levels below 10 kΩ during stimulation. The cathodal electrode was positioned on the right side of the thoracic 10th–12th vertebral levels, and the anode electrode was positioned on the right shoulder (Yamaguchi et al., 2020). We confirmed the Jacoby line (4th lumbar vertebrae), and the vertebrae were counted up from the 4th lumbar vertebrae to the 10th–12th thoracic vertebrae. The electrode was positioned 1.5 cm outside from the 10th–12th thoracic vertebrae. The electrode of the right shoulder was placed on the posterior deltoid muscle, which was confirmed by manipulation. The underlying mechanisms for the effects of tsDCS on the neural plasticity of motor and sensory systems have not been clarified, but previous studies have shown that cathodal tsDCS consistently facilitates the motor cortex and motor-related pathway (Bocci et al., 2014, 2015a,b; Murray et al., 2018; Yamaguchi et al., 2020; Therkidsen et al., 2022). Thus, cathodal tsDCS was adopted in the present study. The stimulation intensity was 2.5 mA (current density was 0.07 mA/cm^2^) for 20 min. Ramp up and ramp down were set for 20 s. The sham tsDCS was delivered at 2.5 mA for 30 s.

#### 2.2.3. Electromyogram recording

The electromyogram (EMG) was recorded using Ag/AgCl electrodes (1 cm diameter, 2 cm inter-electrode distance). The skin was rubbed with an alcohol pad to maintain an electrode impedance of under 5 kΩ throughout the experiment. The EMG electrodes were placed over the right TA muscle belly. The wrap ground electrode for EMG was wrapped just below the knee. EMG signals were recorded at a sampling rate of 5 kHz with 10–1,000 Hz bandpass filtering using Neuropack MEB-2200.

#### 2.2.4. Electroencephalograph recording

The electroencephalograph (EEG) was recorded using Ag/AgCl electrodes (1 cm diameter). The skin was rubbed with an alcohol pad to maintain an electrode impedance of under 5 kΩ throughout the experiment. The active electrode was placed at Cpz representing the somatosensory cortex of the lower extremity, and the reference electrode was placed at the right earlobe (rE), according to the international 10–20 system of electrode placement (Cogiamanian et al., 2008). The wrap ground electrode for EEG was wrapped just below the knee. EEG signals were recorded at a sampling rate of 5 kHz with 0.5–200 Hz bandpass filtering using Neuropack MEB-2200.

### 2.3. Experimental procedure and evaluation

#### 2.3.1. Experiment 1: The combined effects of NMES and tsDCS on MEPs and SICI

**FIGURE 1:**
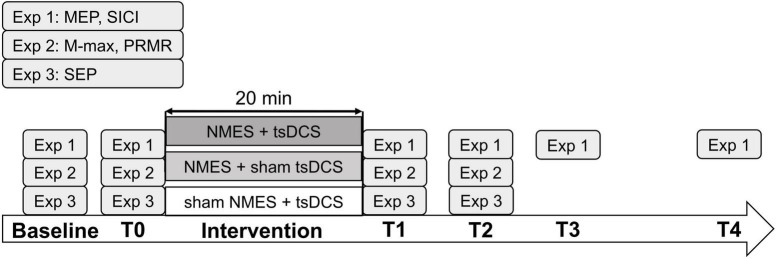
Experimental procedure. The participants received neuromuscular electrical stimulation (NMES) + transcutaneous spinal direct current stimulation (tsDCS), NMES + sham tsDCS, or sham NMES + tsDCS (experiments 1, 2, and 3, respectively) in 20 min session conducted on different days. In experiment 1, motor evoked potentials (MEPs) and short-interval intracortical inhibition (SICI) were measured at baseline, before the intervention (T0), and after the intervention at 0 min (T1), 15 min (T2), 30 min (T3), and 60 min (T4). In experiment 2, maximum M-wave (M-max) and posterior root muscle reflex (PRMR) were measured at baseline, T0, T1, and T2. In experiment 3, somatosensory evoked potentials (SEPs) were measured at baseline, T0, T1, and T2.

Because MEPs were found to increase more after tsDCS was performed with participants lying supine compared with sitting on a chair (Murray et al., 2018), the 24 participants in experiment 1 were instructed to sit on a chair during the assessment and lay supine on a couch during the intervention. To normalize MEPs at each tested time point, MEPs were measured before the assessment (baseline). After the baseline measurement, the MEPs and SICI were assessed just before intervention (T0) and after intervention at 0 min (T1), 15 min (T2), 30 min (T3), and 60 min (T4) (Figure 1).

##### 2.3.1.1. MEP and SICI

To assess corticospinal excitability, a single-pulse TMS was delivered to the left primary motor cortex (M1) responsible for the motor representation of the right TA muscle with a double-cone coil connected to the Magstim 200 (Magstim Co., Whitland, UK). The hotspot of the M1 was confirmed based on the induction of maximum and sustained MEP in the right TA muscle at rest. To stimulate the same hot spot throughout the study, we marked the coil position on the head with a pen. The stimulus intensity was adjusted to 120% of the resting motor threshold (rMT), which was defined as the minimum stimulation intensity over the motor hotspot required to evoke MEPs of no < 50 μV with a 50% probability (Rossi et al., 2009). We averaged 15 measurements of the peak-to-peak MEP amplitude and calculated the mean value among participants. The average MEP value at each tested time point following the intervention was expressed as a percentage of the baseline measurement (%MEPs) and used for statistical analysis.

To assess SICI, we adopt a sub-threshold conditioning paired-pulse paradigm (Kujirai et al., 1993). We used 80% of the active motor threshold (aMT) for the conditioning stimulation and 120% of the rMT for the test stimulus (Yamaguchi et al., 2012). The aMT was defined as the minimum stimulus intensity required to evoke MEPs of >200 μV with a 50% probability during isometric contraction upon 100 μV EMG of the right TA muscle (Yamaguchi et al., 2018; Katagiri et al., 2020). Participants were instructed to maintain isometric contraction with visual feedback of the EMG amplitude. The test stimulus intensity was adjusted to maintain the average amplitude recorded before each intervention throughout the experiment. The interstimulus interval (ISI) controlled by LabVIEW software (National Instruments Co., TX, USA) was 2.5 ms, and 15 trials were recorded for each ISI and test stimulation (Katagiri et al., 2020). The SICI amplitudes were expressed as percentages of the mean test MEPs amplitudes and used for statistical analysis.

#### 2.3.2. Experiment 2: The combined effect of NMES and tsDCS on PRMR

The 15 participants enrolled in experiment 2 were instructed to lay supine on a couch during the experiment. We measured the PRMR before the assessment (baseline), and the PRMR and maximum M-wave (M-max) were measured before intervention (T0), immediately after (T1), and 15 min after the stimulation (T2) (Figure 1). From our pilot study that investigated the lasting effects of combined stimulation on PRMR and M-wave, the effects did not last > 15 min after the stimulation.

##### 2.3.2.1. PRMR

To induce the PRMR from the TA muscle, a stimulus (duration: 1.0 ms) was delivered at 0.3 Hz to the thoracic 10–12 vertebral levels of the posterior root *via* two electrodes connected to Neuropack MEB-2200. The cathodal electrode (5 cm × 7 cm) was positioned on the right side of thoracic 10–12 vertebral levels, and the anode electrode (10 cm × 10 cm) was positioned on the trunk above the umbilicus (Minassian et al., 2007; Milosevic et al., 2019). The stimulus intensity was adjusted to 120% of the MT, which was defined as the minimum stimulation intensity required to evoke a sustained PRMR of 100 μV in the TA muscle. We obtained five measurements of the peak-to-peak amplitude of the PRMR and calculated the mean value among participants at each stimulus intensity. The PRMR amplitudes were expressed as a percentage of the mean M-max amplitudes (%PRMR) and used for statistical analysis.

##### 2.3.2.2. M-wave

To induce the M-wave from the right TA muscle, the stimulus (duration: 0.2 ms) was delivered at 0.3 Hz to the CPN by bipolar stimulus electrodes (1.5 cm diameter, 3.0 cm inter-electrode distance) connected to Neuropack MEB-2200. A supra-maximal stimulus intensity was used to evoke M-max. The peak-to-peak amplitude of M-max was measured.

#### 2.3.3. Experiment 3: The combined effect of NMES and tsDCS on SEPs

The 16 participants in experiment 3 were instructed to lay supine on a couch during the intervention. To normalize SEPs at each time point, SEPs were measured before the assessment (baseline). After the baseline measurement, SEPs were assessed just before the intervention (T0) and after the intervention at 0 min (T1) and 15 min (T2) (Figure 1). Based on our pilot study, we decided that SEPs required to be assessed until 15 min after the stimulation.

##### 2.3.3.1. SEP

**FIGURE 2:**
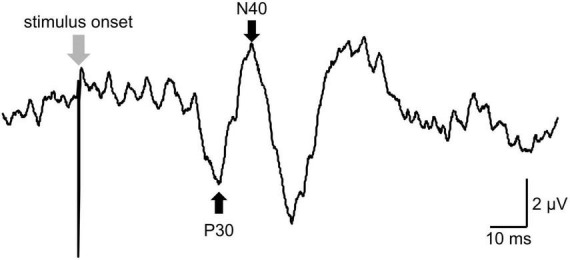
The raw data of somatosensory evoked potentials (SEPs). The peaks of the SEPs were labeled based on latency from the stimulus onset for peak-to-peak analysis. P30 was defined as a positive peak with a latency of around 39 ms (39 ± 5 ms). N40 was defined as a negative peak following P30 and with a latency of around 45 ms (45 ± 5 ms).

Somatosensory evoked potentials were evoked by stimulation of the right posterior tibialis nerve at the ankle. In total, 300 pulses of 0.2 ms duration were delivered at a rate of 3 Hz (Cogiamanian et al., 2008). The stimulus intensity was adjusted to 300% of the perceptual threshold, defined as the minimum stimulus intensity that can be perceived by participants (Cruccu et al., 2008). Bipolar stimulus electrodes (the cathode electrode was located 3.0 cm proximal to the anode electrode) were connected to Neuropack MEB-2200. To record S1 excitability, recording electrodes were placed over the Cpz referred to rE. The peaks of the SEPs were labeled based on latency from the stimulus onset (Schabrun et al., 2012; Figure 2). SEPs were analyzed as peak-to-peak amplitudes for the component of N30-P40 (Cogiamanian et al., 2008). SEPs waveforms were recorded 300 times, and the mean value of the peak-to-peak SEPs amplitude among participants was calculated. The average SEPs value at each time point following the intervention was expressed as a percentage of the baseline measurement (%SEPs) and used for statistical analysis.

### 2.4. Statistical analysis

The Shapiro–Wilk test was used to determine whether the baseline MEPs, %MEPs, SICI, baseline PRMR, %PRMR, M-max, baseline SEPs, and %SEPs were normally distributed. For experiment 1, a mixed-effects model for the repeated-measures analysis of variance (MMRM) was used to determine the effects of time (T0, T1, T2, T3, and T4) or condition (NMES + tsDCS, NMES + sham tsDCS, and sham NMES + tsDCS) on the %MEPs and SICI data. For experiments 2 and 3, a MMRM was used to determine the effects of time (T0, T1, and T2) or condition (NMES + tsDCS, NMES + sham tsDCS, and sham NMES + tsDCS) on the %PRMR, M-max, and %SEPs data. When a significant main effect was observed, we performed *post-hoc* comparisons. We used paired *t*-tests with Bonferroni adjustments for normally distributed data, and the Wilcoxon signed-rank test with Bonferroni adjustments was used for non-normally distributed data. Statistical significance was set at *P* < 0.05 for all comparisons. All statistical analyses were performed using SPSS 24 (IBM, Armonk, NY, United States).

## 3. Results

The Shapiro–Wilk test confirmed that only SICI values were normally distributed; therefore, they were analyzed using a parametric test. The data of baseline MEPs, PRMR, M-max, and SEPs are described in the Supplementary material. There was no significant difference in any baseline data among all conditions (*P* > 0.05). After each task, a questionnaire evaluated whether the participants felt the combined stimulation or sham stimulation. The chi-square (x2) test was used to confirm the number of correct responses. No significant differences were found in the number of correct responses to the stimulus questionnaire in the NMES + sham tsDCS condition (*P* = 0.611) and the sham NMES + tsDCS condition (*P* = 0.563) compared with the number of correct answers in the combined stimulation condition.

### 3.1. Experiment 1: The combined effects of NMES and tsDCS on MEPs and SICI

**FIGURE 3:**
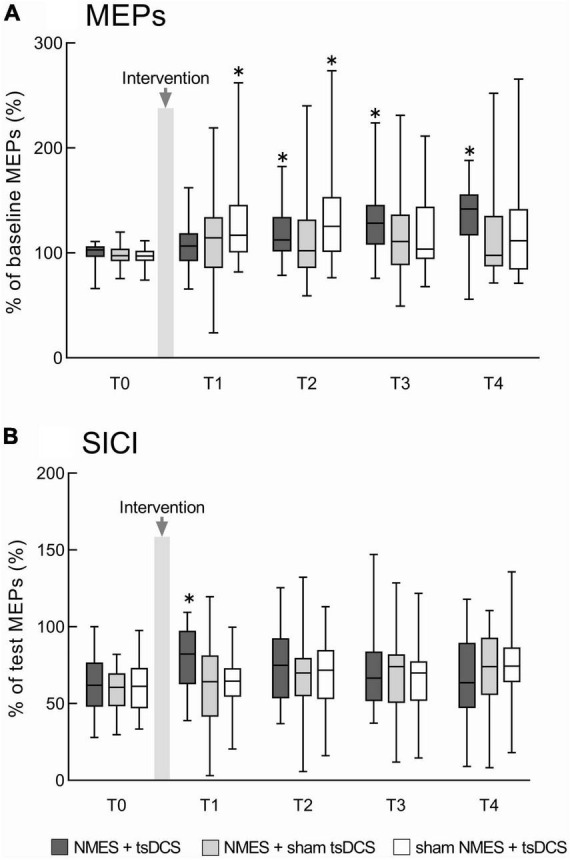
The combined effects of neuromuscular electrical stimulation (NMES) and transcutaneous spinal direct current stimulation (tsDCS) on motor evoked potentials (MEPs) and short-interval intracortical inhibition (SICI). **(A)** Represents the MEPs changes before and after the intervention. MEP amplitudes were normalized to baseline MEP amplitudes. **(B)** Represents the SICI changes before and after the intervention. Conditioned MEP amplitudes were normalized to test MEP amplitudes to calculate SICI. NMES + tsDCS is denoted as dark gray boxes, NMES + sham tsDCS is denoted as light gray boxes, and sham NMES + tsDCS is denoted as white boxes. The median value and interquartile ranges are represented by horizontal lines within boxes and whiskers representing minimum and maximum values, respectively. Asterisks indicate significant differences compared to “T0” (*P* < 0.05).

There were significant main effects of time on %MEPs in the NMES + tsDCS (MMRM, *F*_4, 92_ = 10.501, *P* = 0.001) and sham NMES + tsDCS (MMRM, *F*_4, 92_ = 5.242, *P* = 0.001) conditions. However, there were no significant main effects of time on %MEPs in the NMES + sham tsDCS condition (*F*_4, 92_ = 2.023, *P* = 0.098) or among all conditions (MMRM, *F*_2, 46_ = 1.968, *P* = 0.151). Compared to pre %MEPs, analysis using the Wilcoxon signed-rank test with Bonferroni adjustment showed that NMES + tsDCS significantly increased %MEPs at T2 (*P* = 0.040), T3 (*P* = 0.010), and T4 (*P* = 0.001), and sham NMES + tsDCS significantly increased %MEPs at T1 (*P* = 0.010) and T2 (*P* = 0.020). These results indicate that the combined stimulation of NMES and tsDCS increased corticospinal excitability for 60 min or more (Figure 3A).

There was no significant interaction between time and condition for SICI (MMRM, *F*_8, 322_ = 1.355, *P* = 0.216). Time had a main effect on SICI in the NMES + tsDCS condition (MMRM, *F*_4, 92_ = 3.388, *P* = 0.012), while there were no main effects of time on SICI in any of the other conditions (MMRM, NMES + sham tsDCS: *F*_4, 92_ = 2.406, *P* = 0.055; sham NMES + tsDCS: *F*_4,92_ = 2.264, *P* = 0.068). There was a significant main effect of condition on SICI at T1 (MMRM, *F*_2, 46_ = 3.932, *P* = 0.027), but these effects were not present for any other time point (MMRM, T0: *F*_2, 46_ = 0.506, *P* = 0.606; T2: *F*_2,46_ = 0.395, *P* = 0.676; T3: *F*_2,46_ = 0.116, *P* = 0.891; T4: *F*_2,46_ = 0.768, *P* = 0.462). Compared to T0 SICI, analysis using the *t*-test with Bonferroni adjustments revealed that NMES + tsDCS significantly decreased SICI at T1 only (*P* = 0.010), while there were no significant differences in any other time point (T2: *P* = 0.163; T3: *P* = 0.769; T4: *P* > 0.999). Compared to SICI in the NMES + tsDCS condition at T0, there were no significant differences in the NMES + sham tsDCS condition (*P* = 0.056) or the sham NMES + tsDCS condition (*P* = 0.059). These results indicate that combined stimulation of NMES and tsDCS decreases the intracortical inhibition immediately after stimulation (Figure 3B). Individual raw data for MEPs and SICI amplitudes have been provided in the Supplementary material.

### 3.2. Experiment 2: The combined effect of NMES and tsDCS on PRMR

**FIGURE 4:**
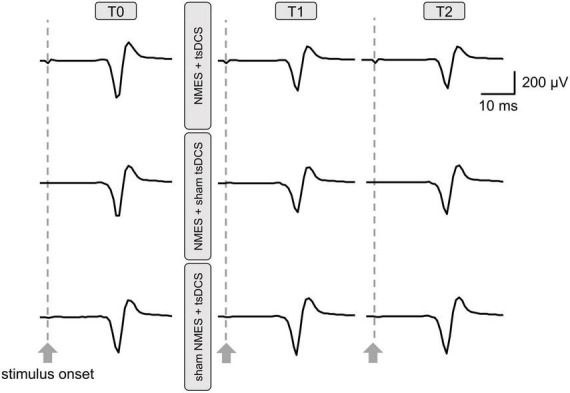
Raw traces of posterior root muscle reflex (PRMR) waveforms from the right tibialis anterior muscle were obtained from a participant before and after neuromuscular electrical stimulation (NMES) + transcutaneous spinal direct current stimulation (tsDCS); NMES + sham tsDCS; and sham NMES + tsDCS. Each waveform represents the average of five trials.

Figure 4 presents the PRMR waveforms from the right TA muscle. There was a significant main effect of time on %PRMR in the NMES + tsDCS condition (MMRM, *F*_2, 28_ = 3.779, *P* = 0.035), but this effect did not exist in the other conditions (MMRM, NMES + sham tsDCS: *F*_2, 28_ = 0.816, *P* = 0.453; sham NMES + tsDCS: *F*_2, 28_ = 3.148, *P* = 0.058). Moreover, there were no significant main effects of condition on %PRMR in all time points (MMRM, T1: *F*_2, 28_ = 0.533, *P* = 0.593; T2: *F*_2, 28_ = 0.361, *P* = 0.700). Comparing to T0%PRMR using Wilcoxon signed rank test with Bonferroni adjustments, we found that NMES + tsDCS significantly decreased the %PRMR at T1 only (*P* = 0.018), while there was no significant difference at T2 (*P* = 0.234). These results indicate that combined stimulation of NMES and tsDCS decreased spinal reflex excitability immediately after stimulation (Table 1).

**TABLE 1:** Changes in %PRMR before and after the combined stimulation of neuromuscular electrical stimulation (NMES) and transcutaneous spinal direct current stimulation (tsDCS).

	T0	T1	T2
NMES + tsDCS	13.22 (5.27)	11.61* (5.54)	12.13 (6.58)
NMES + sham tsDCS	12.34 (7.81)	10.30 (3.58)	10.70 (3.56)
sham NMES + tsDCS	12.43 (7.77)	11.72 (7.66)	11.07 (8.06)

The values represent the mean (standard deviation). Posterior root muscle reflex (PRMR) values represent the percentage of normalized maximum M-wave (M-max) values to calculate %PRMR. Asterisks indicate a significant difference compared to “T0” (*P* < 0.05).

There was a significant main effect of time on M-max in the sham NMES + tsDCS condition (MMRM, *F*_2, 28_ = 3.428, *P* = 0.047), which was not present in the other conditions (MMRM, NMES + tsDCS: *F*_2, 28_ = 1.340, *P* = 0.278; NMES + sham tsDCS: *F*_2, 28_ = 1.198, *P* = 0.317). Additionally, analysis with the Wilcoxon signed rank test with Bonferroni adjustments revealed no significant differences in M-max between T0 and any other time point (T1: *P* = 0.573; T2: *P* = 0.264). The average of raw data of the M-max amplitudes is provided in Table 2.

**TABLE 2:** Changes in M-max before and after combined stimulation using neuromuscular electrical stimulation (NMES) and transcutaneous spinal direct current stimulation (tsDCS).

	T0	T1	T2
NMES + tsDCS	2.50 (0.86)	2.64 (0.87)	2.56 (1.00)
NMES + sham tsDCS	2.84 (1.11)	3.00 (1.43)	3.02 (1.41)
sham NMES + tsDCS	2.87 (1.07)	2.95 (1.00)	3.05 (0.95)

The values represent the mean (standard deviation) of the maximum M-wave (M-max) (mV).

### 3.3. Experiment 3: The combined effect of NMES and tsDCS on SEPs

There were no significant main effects of time on SEPs (MMRM, NMES + tsDCS: *F*_2, 30_ = 1.385, *P* = 0.266; NMES + sham tsDCS: *F*_2, 30_ = 1.766, *P* = 0.188; sham NMES + tsDCS: *F*_2, 30_ = 2.055, *P* = 0.146) or of condition on SEPs (MMRM, T1: *F*_2, 30_ = 1.043, *P* = 0.365; T2: *F*_2, 30_ = 0.740, *P* = 0.486). These results indicate that combined stimulation of NMES and tsDCS do not affect somatosensory cortical excitability. The average of raw data for the SEP amplitudes is provided in Table 3.

**TABLE 3:** Changes in %SEPs before and after combined stimulation using neuromuscular electrical stimulation (NMES) and transcutaneous spinal direct current stimulation (tsDCS).

	T0	T1	T2
NMES + tsDCS	108.3 (19.4)	110.8 (31.4)	121.9 (42.2)
NMES + sham tsDCS	104.1 (18.1)	110.8 (27.5)	119.9 (35.7)
sham NMES + tsDCS	95.3 (20.0)	97.9 (31.4)	111.0 (44.5)

The values represent the mean (standard deviation). Somatosensory evoked potential (SEP) values represent the percentage of baseline SEP values to calculate %SEPs.

## 4. Discussion

This study demonstrates that combining NMES and tsDCS increases corticospinal excitability of the TA muscle and prolongs motor cortex excitability for ≥60 min. Intercortical inhibition and spinal reflex excitability decreased, and there was no change in somatosensory cortex excitability, immediately after the combined stimulation. These results provide evidence of the underlying mechanism of combining NMES with tsDCS to enhance the primary motor cortex excitability, which may contribute to the development of neurorehabilitation for CNS lesions.

Applying NMES to peripheral afferent fibers increases the motor cortex excitability *via* cortico-cortical projections from the somatosensory cortex in humans and animals (Kaneko et al., 1994a,b; Hamdy et al., 1998; Ridding et al., 2000; Kaelin-Lang et al., 2002; Khaslavskaia et al., 2002; Luft et al., 2002; Knash et al., 2003; Taib et al., 2005; Mang et al., 2010; Chipchase et al., 2011; Schabrun et al., 2012; Yamaguchi et al., 2012; Sasaki et al., 2017; Carson and Buick, 2021; Insausti-Delgado et al., 2021). Meanwhile, tsDCS over the lumber spinal segments activates afferent fibers at the spinal dorsal root and increases motor cortex excitability (Bocci et al., 2015a,b; Murray et al., 2018; Yamaguchi et al., 2020; Therkidsen et al., 2022). However, in agreement with a previous study (Cogiamanian et al., 2008), the results of experiment 3 showed that somatosensory cortex excitability was not changed following either tsDCS alone or combined stimulation. A possible reason was that afferent inputs with tsDCS increase motor cortex excitability *via* the projection of thalamic neurons to the motor cortex, which is a part of the transcortical reflex pathway (Asanuma et al., 1980; Christensen et al., 2000; Yamaguchi et al., 2020; Therkidsen et al., 2022). Such different afferent pathways (e.g., the cortico-cortical pathway from the somatosensory cortex and transcortical reflex pathway) activated by NMES and tsDCS may provide a summation effect, thereby enhancing the motor cortex excitability and inducing neural plasticity of the primary motor cortex. In addition, a previous study reported that cutaneous stimulation to the sural nerves has a facilitating effect on the TA MEPs elicited with TMS (Wolfe and Hayes, 1995). Thus, the influence of the combined stimulation *via* cutaneous nerves on the corticospinal projection requires investigation. Moreover, the combined stimulation decreased the SICI, which reflects motor cortex inhibitory interneuron excitability *via* GABA_*A*_ receptors (Ziemann et al., 2015). A previous study reported that higher intensity electrical stimulation increases activation of afferent fibers and decreases intracortical inhibition (Golaszewski et al., 2012). Thus, plastic changes in the motor cortex excitability may be induced by increasing afferent inputs to the motor cortex *via* the combined stimulation. On the contrary, there was variability in MEPs and SICI after each intervention. A previous study reported the variability effects of tsDCS because the electric field varies depending on individual differences in tissue and anatomical characteristics, such as the thickness of the soft tissue between the participant’s skin and the dorsal root of the spinal cord (Yamaguchi et al., 2020).

Interestingly, experiment 1 revealed that corticospinal excitability was increased 15 min after the combined stimulation of NMES and tsDCS, but the increased corticospinal excitability was not observed immediately after the intervention. This may be because the combined stimulation of NMES and tsDCS decreased the spinal motor neuron’s excitability, making it difficult to detect changes in the corticospinal excitability immediately after the stimulation. This speculation is supported by the results of experiment 2, which showed that excitability in the spinal motor neuron pool was decreased in the combined stimulation only. The spinal motor neuron’s excitability is influenced by transmission in inhibitory pathways, such as Ib inhibition mediated by Ib inhibitory interneurons and recurrent inhibition mediated by Renshaw cells (Pierrot-Deseilligny and Mazevet, 2000; Hultborn, 2006). Therefore, these inhibitory pathways might be activated by the combined stimulation, which could decrease the spinal motor neuron’s excitability immediately after stimulation. Another possibility is neural or muscle fatigue (Stephens and Taylor, 1972), but the M-max amplitudes were unchanged in experiment 2. In agreement with a previous study (Murray et al., 2018), we found that spinal excitability shows a decreasing trend after the cathodal tsDCS with sham NMES. Therefore, cathodal tsDCS may affect the spinal inhibitory circuits. In addition, the time sequence of NMES and tsDCS may be important to induce neural plasticity. Many studies have reported the effects of stimulus timing on the efficacy of interventions that can induce plasticity (Iyer et al., 2003; Ridding and Ziemann, 2010; Yamaguchi et al., 2018). Thus, further investigation is required to examine the interaction of stimulus timing between NMES and tsDCS on neural plasticity. Further studies are necessary to identify the effects of NMES and tsDCS on spinal inhibitory pathways and other neural circuits related to the excitability of the spinal motor neurons. It is also interesting to optimize the parameter of the combined stimulation to increase the plasticity of the spinal motor neuron pool.

In this study, both the motor and somatosensory cortex excitability were unchanged by NMES alone. NMES-induced changes in sensorimotor cortex activity have a dose–response relationship with the recruitment of afferent fibers, which was influenced by stimulation intensity (Smith et al., 2003; Cho et al., 2007; Insausti-Delgado et al., 2021) and duration (Chipchase et al., 2011). Thus, 20 min of NMES with MT stimulus intensity might be insufficient to modulate the sensorimotor cortex excitability. On the other hand, in agreement with previous studies (Bocci et al., 2015a; Murray et al., 2018; Yamaguchi et al., 2020), tsDCS alone increased the motor cortex excitability up to 15 min after the stimulation, but the lasting effect disappeared after 30 min. Therefore, increasing afferent inputs by different stimulus positions (i.e., peripheral nerve and spinal dorsal root) may be an important factor for inducing neural plasticity of the motor cortex.

Many studies reported that the enhancement of plastic changes in the motor cortex plays a crucial role in improving motor function after stroke and spinal cord injury (Jurkiewicz et al., 2007; Christiansen and Perez, 2018). Our finding provides the possibility that tsDCS enhances the recovery of motor function after CNS lesions by NMES alone (Sharififar et al., 2018). In addition, a previous study reported that the ankle dorsiflexion strength affected the gait velocity of patients with stroke (Lin et al., 2006). Thus, the combined stimulation of NMES and tsDCS may be an adjuvant therapy for motor rehabilitation. For example, it is possible to use this method for locomotor training, such as treadmill walking with partial body weight support (Mehrholz et al., 2017), robot-assisted locomotor training (Hornby et al., 2008), or pedaling exercise (Yamaguchi et al., 2013). However, lower extremity muscle activity during gait is also influenced by other descending pathways (e.g., the reticulospinal tract) (Soulard et al., 2020). Thus, the combined effect on gait exercise requires careful consideration of the patients.

The combined stimulation of NMES and tsDCS can be effectively used to increase motor cortex excitability of the TA muscle in rehabilitation programs of patients with CNS injuries. However, this study has some limitations. First, our study was conducted in healthy participants. Second, the participants were all male. A previous study reported that transcranial direct current stimulation (tDCS) could modulate neural activity more in females than in males (Boggio et al., 2008). Thus, sex differences may also influence the effect of combined stimulation, but as with tDCS, women may have a better response. Therefore, the combined effects of NMES and tsDCS on motor dysfunction should be examined in patients with CNS injury and in women. Third, inter-participant variability in responses to each stimulation is possible. Therefore, further studies are needed to reduce variability such as in stimulus parameters and differences in anatomical characteristics. Finally, no significant difference was observed in the number of correct answers between each condition (real or sham), but participants might have noticed the stimulation condition during the stimulation because the stimulus intensity of the NMES was applied above the motor threshold in the real condition.

In conclusion, cathodal tsDCS enhances the facilitation effects of NMES on the corticospinal projection, indicating that incremental ascending volleys to the primary motor cortex *via* the stimulation of the peripheral nerve and spinal dorsal roots play an important role in motor cortical plasticity. The current findings provide evidence for a new neuromodulation method to promote neural plasticity and motor functional recovery after stroke and spinal cord injury. Further studies are warranted to clarify the clinical application of the current approach in patients with neurological motor dysfunction.

## Data availability statement

The raw data supporting the conclusions of this article will be made available by the authors, without undue reservation.

## Ethics statement

The studies involving human participants were reviewed and approved by the Ethics Committee of Yamagata Prefectural University Health of Sciences. The patients/participants provided their written informed consent to participate in this study.

## Author contributions

TK and TY conceived and designed the experiments and drafted the manuscript. TK recruited participants and collected and analyzed data. TK, DK, KY, MN, KT, MJ, TS, HK, and TY interpreted the results of the experiments. ST constructed the program for data collection. All authors approved the final version of the submitted manuscript.
