# Supplementary material for: Combined neuromuscular electrical stimulation and transcutaneous spinal direct current stimulation increases motor cortical plasticity in healthy humans
Source: Front Neurosci. 2023 Jan 13;16:1034451. doi: 10.3389/fnins.2022.1034451 (PMC10115158; doi:10.3389/fnins.2022.1034451)
Supplement: Supplementary file 1 [file Data_Sheet_1.docx]

***Supplementary Material***

**Supplemental table**

The data of baseline motor evoked potentials (MEPs), posterior root muscle reflex (PRMR), maximum M-wave (M-max), and somatosensory evoked potentials (SEPs) are represented in the table. There were no significant differences between these baseline conditions (*P* >0.05).

|  | NMES + tsDCS | NMES + sham tsDCS | sham NMES + tsDCS | F-value | P-value |
| --- | --- | --- | --- | --- | --- |
| MEPs (µV) | 316 ± 227 | 346 ± 252 | 283 ± 204 | 1.24 | 0.298 |
| PRMR (µV) | 300 ± 89 | 314 ± 121 | 324 ± 210 | 0.26 | 0.772 |
| M-max (mV) | 2.50 ± 0.86 | 2.84 ± 1.11 | 2.87 ± 1.07 | 2.11 | 0.140 |
| SEPs (µV) | 2.98 ± 2.28 | 2.96 ± 2.52 | 3.27 ± 2.68 | 1.34 | 0.277 |

The data represent the mean ± SD (standard deviation). Abbreviations; MEPs: motor evoked potentials, PRMR: posterior root muscle reflex, M-max: maximum M-wave, SEPs: somatosensory evoked potentials, SD: standard deviations


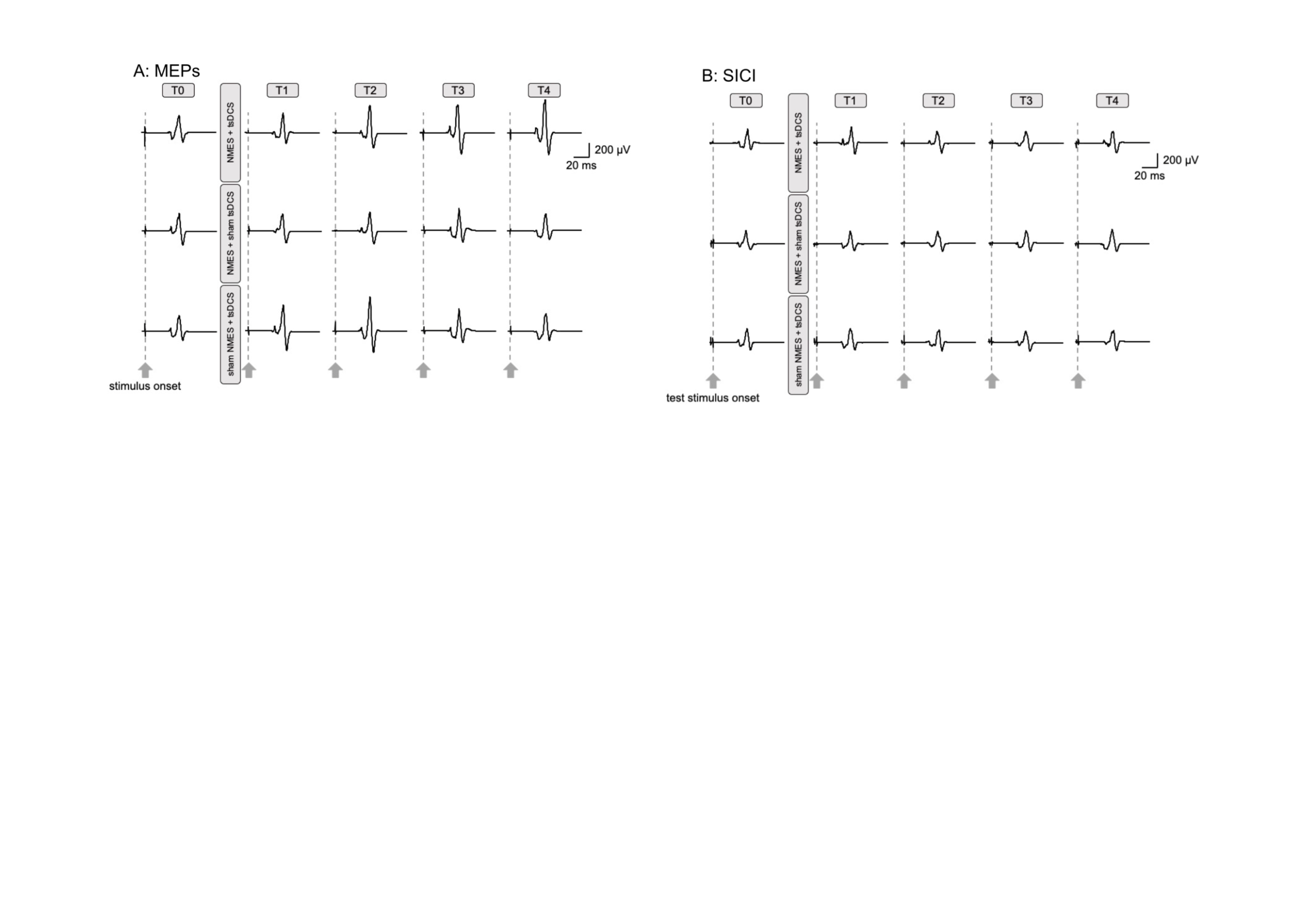


**Supplemental Figure. Individual raw data for MEPs and SICI amplitudes.**

Individual raw data for motor evoked potentials (MEPs) (A) and short-latency intracortical inhibition (SICI) (B) waveforms of a participant before and after neuromuscular electrical stimulation (NMES) + transcutaneous spinal direct current stimulation (tsDCS); NMES + sham tsDCS; and sham NMES + tsDCS.
